# Structural insight into the novel *Thermus thermophilus* SPOUT methyltransferase RlmR catalysing Um2552 formation in the 23S rRNA A-loop: a case of convergent evolution

**DOI:** 10.1093/nar/gkaf432

**Published:** 2025-05-30

**Authors:** Yousra Tanouti, Martine Roovers, Philippe Wolff, Antony Lechner, Dany Van Elder, André Feller, Romuald Soin, Cyril Gueydan, Véronique Kruys, Louis Droogmans, Geoffray Labar

**Affiliations:** Labiris, Avenue Emile Gryson 1, B-1070 Bruxelles, Belgium; Laboratoire de Biologie Moléculaire du Gène, Institut de Biologie et de Médecine Moléculaires, Université Libre de Bruxelles (ULB), Rue des Professeurs Jeener et Brachet 12, B-6041 Gosselies, Belgium; Labiris, Avenue Emile Gryson 1, B-1070 Bruxelles, Belgium; Architecture et Réactivité de l’ARN, Institut de Biologie Moléculaire et Cellulaire du CNRS, Université de Strasbourg, F-67084 Strasbourg, France; Architecture et Réactivité de l’ARN, Institut de Biologie Moléculaire et Cellulaire du CNRS, Université de Strasbourg, F-67084 Strasbourg, France; Laboratoire de Chimie Biologique, Université Libre de Bruxelles (ULB), Labiris, Avenue Emile Gryson 1, B-1070 Bruxelles, Belgium; Laboratoire de Chimie Biologique, Université Libre de Bruxelles (ULB), Labiris, Avenue Emile Gryson 1, B-1070 Bruxelles, Belgium; Laboratoire de Biologie Moléculaire du Gène, Institut de Biologie et de Médecine Moléculaires, Université Libre de Bruxelles (ULB), Rue des Professeurs Jeener et Brachet 12, B-6041 Gosselies, Belgium; Laboratoire de Biologie Moléculaire du Gène, Institut de Biologie et de Médecine Moléculaires, Université Libre de Bruxelles (ULB), Rue des Professeurs Jeener et Brachet 12, B-6041 Gosselies, Belgium; Laboratoire de Biologie Moléculaire du Gène, Institut de Biologie et de Médecine Moléculaires, Université Libre de Bruxelles (ULB), Rue des Professeurs Jeener et Brachet 12, B-6041 Gosselies, Belgium; Laboratoire de Chimie Biologique, Université Libre de Bruxelles (ULB), Labiris, Avenue Emile Gryson 1, B-1070 Bruxelles, Belgium; Labiris, Avenue Emile Gryson 1, B-1070 Bruxelles, Belgium

## Abstract

The A-loop of the 23S ribosomal RNA is a critical region of the ribosome involved in stabilizing the CCA-end of A-site-bound transfer RNA. Within this loop, nucleotide U2552 is frequently 2′-*O*-methylated (Um2552) in various organisms belonging to the three domains of life. Until now, two enzymatic systems are known to modify this position, relying on either a Rossmann fold-like methyltransferase (RFM) or a small RNA-guided system. Here, we report the identification of a third system involved in Um2552 formation, consisting of a methyltransferase of the SPOUT (SpoU-TrmD) superfamily encoded by the *ttc1712* open reading frame of *Thermus thermophilus*, herein renamed RlmR. In *Escherichia coli* and human mitochondria, the absence of the RFM enzyme responsible for Um2552 formation is known to cause severe defects in ribogenesis and ribosome function. In contrast, no comparable effect was observed upon *ttc1712* gene invalidation in *T. thermophilus*. We also report the high-resolution crystal structure of RlmR in complex with a 59-mer substrate RNA. The structure highlights significant conformational rearrangements of the A-loop and provides a new insight into the catalytic mechanism, revealing structural features that may be generalized to other SpoU methyltransferases.

## Graphical Abstract

**Graphical Abstract:**
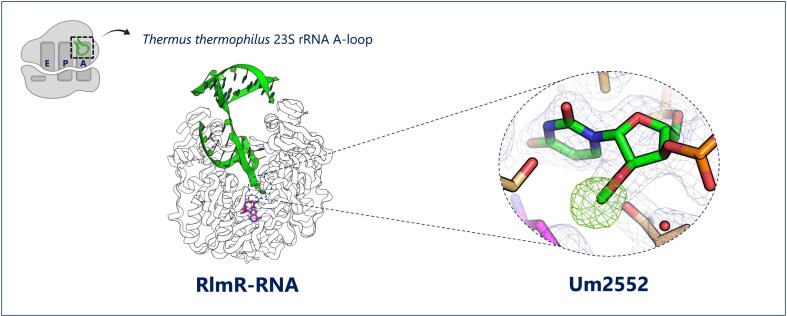

## Introduction

Ribosomes are conserved ribonucleoprotein complexes dedicated to protein synthesis. Bacterial and eukaryotic ribosomes differ in their size, ribosomal RNA (rRNA) length, and number of associated proteins. However, their core structure shares striking similarities [1].

Specific nucleotides of rRNA undergo chemical modifications, attached to either the ribose or the base. Rather than being randomly scattered across the ribosome, these modifications are present in functional parts of this machinery, like the decoding center, the peptidyl transferase center, as well as the A- and P-tRNA (transfer RNA) binding sites. This specific distribution underlines their overall biological importance [2]. Despite this modification clustering, all organisms display a distinct repertoire of methylated rRNA nucleotides, which is consistent with the phylogenetic distribution of RNA methyltransferases (MTases) across bacterial phyla [3, 4].

One of the most abundant rRNA modifications is 2′-*O*-methylation, which can be formed through several enzymatic pathways [5, 6]. In bacteria, this modification is catalyzed by MTases belonging to Class I, which contain a Rossmann fold-like (called RFM for Rossmann fold-like methyltransferase), or to Class IV (called SPOUT for SpoU-TrmD). SPOUT MTases are divided into the SpoU (renamed TrmH) and TrmD families, based on the two founding members of this superfamily. SpoU and TrmD members catalyze, respectively, ribose and base modification. Recently, a more thorough phylogenetic analysis of the SPOUT superfamily led to a subdivision into four major clades [7]. Despite this diversity, all members share a distinctive trefoil-knotted domain in their tertiary structure. In eukaryotes and archaea, besides the stand-alone enzymes, RNA MTases can be associated with other proteins and guided to their target RNA by a small RNA [8, 9].

Among the modifications found in the large ribosomal subunit, the 2′-*O*-methylation of U2552 (*Escherichia coli* numbering)—a conserved nucleotide within the 23S rRNA A-loop—has been reported in species across all domains of life [10–14]. U2552 is located in the peptidyl transferase centre, adjacent to G2553, a critical base that anchors the CCA terminus of tRNA within the large subunit [15].

To date, enzymes from two different clusters of orthologous groups (COGs) were reported to catalyze Um2552 formation. They correspond to either a stand-alone enzyme of the RFM family (COG0293: RlmE in *E. coli*, formerly FtsJ, RrmJ; MRM2 in human and yeast mitochondria) [11, 12, 14], or a small nucleolar RNA-guided enzyme (COG1889: snR52-Nop1 in yeast) [16, 17]. This case of convergent evolution, in which different systems are involved in a specific RNA modification in prokaryote and eukaryote, is not unique. For instance, the m^1^G37 and Ψ55 modifications in tRNA are catalyzed by enzymes from different COGs, depending on the domain of life [18–21].

The widespread occurrence of Um2552 across the three domains of life underscores the functional significance of this modification. Indeed, RlmE-mediated methylation triggers late steps of 50S subunit assembly in *E. coli*, making RlmE a crucial actor in ribogenesis [22, 23]. In line with this, RlmE deficiency leads to a severe growth defect, an accumulation of 45S assembly intermediate, and an increased sensibility to various antibiotics [22, 24–25]. The importance of enzymes modifying U2552 is also well documented in the case of human and yeast mitochondria [12, 14, 26–28].

A 2′-*O*-methyluridine is also found at position 2563 in the 23S rRNA of *Thermus thermophilus*, which corresponds to position 2552 in *E. coli* [29, 30]. Intriguingly, no RlmE homolog is present among the Deinococci, the class of bacteria to which *T. thermophilus* belongs [4]. Instead, RlmE homologs are exclusively found in various bacteria belonging to the α, β, and γ Proteobacteria, Spirochaetia, and in certain δ Proteobacteria [4]. The enzyme responsible for the formation of Um2552 in the 23S rRNA of *T. thermophilus* thus remains to be identified. In this paper, we report the identification of this enzyme as well as its crystal structure in complex with RNA.

## Materials and methods

### General procedures

Ampicillin was used at a concentration of 50 μg/ml and kanamycin at 30 μg/ml. Restriction endonucleases, Phusion DNA polymerase, and T4 DNA ligase were purchased from Thermo Fisher Scientific. [Methyl-^14^C] *S*-adenosylmethionine ([methyl-^14^C]-SAM) and [methyl-^3^H]-SAM were from PerkinElmer. Nuclease P1 was from Sigma. Site-directed mutagenesis was performed using the QuikChange Kit (Agilent).

### Cloning of the *T. thermophilus ttc1712* open reading frame

A synthetic *T. thermophilus ttc1712* open reading frame (ORF) respecting the codon usage of *E. coli* was synthetically generated (GeneArt, Thermo Fisher Scientific). The sequence of *ttc1712* was flanked by the restriction sites NdeI and XhoI to facilitate cloning into the pET28 expression vector. This plasmid allows T7 expression in *E. coli* of the *T. thermophilus* TTC1712 protein bearing an N-terminal His-tag. TTC1712 variants were generated using site-directed mutagenesis of the *tt1712* gene.

### Plasmid construction for *ttc1712* gene inactivation in *T. thermophilus* HB27

Oligonucleotides TTC1712-1 and TTC1712-2 (Supplementary Table S1) were used to amplify the *ttc1712* gene from *T. thermophilus* genomic DNA using Phusion DNA polymerase. The obtained 783 bp polymerase chain reaction (PCR) fragment was cloned in the pJET2.1 vector. To allow a replacement of part of the *ttc1712* gene by a fragment encoding the thermoresistant form of the kanamycin nucleotidyltransferase (Km^r^) [31], a second BamHI site was created in the *ttc1712* gene by site directed mutagenesis using oligonucleotides TTC1712Bam-1 and -2 (Supplementary Table S1). BamHI restriction of the resulting plasmid generated a 228 bp BamHI–BamHI fragment, which was then replaced by the 890 bp Km^r^ BamHI–BamHI fragment. The latter fragment was obtained by PCR amplification using the pML11 template [32], Phusion DNA polymerase, and oligonucleotides Km-1 and Km-2 (Supplementary Table S1), both bearing a BamHI restriction site.

### Inactivation of ttc1712 in *T. thermophilus*

The *ttc1712* gene in *T. thermophilus* was inactivated through the insertion of a kanamycin resistance cassette via homologous recombination. The transformation was performed as described in [33].

### Mutation and T7 *in vitro* transcription of the *T. thermophilus* 23S rRNA gene

The *ttc3035* gene encoding 23S rRNA was amplified from *T. thermophilus* genomic DNA using Phusion DNA polymerase and the oligonucleotides Th23S-1 and -2 (Supplementary Table S1) and cloned in the pJET2.1 vector. The T2552A, T2552C, and T2552G mutants were generated by site-directed mutagenesis using oligonucleotides listed in Supplementary Table S1. T7 *in vitro* transcription was performed according to the instructions of the RiboMAX Kit (Ambion). The 23S rRNA transcripts were purified using illustra MicroSpin G25 columns (GE Healthcare).

### Expression and purification of TTC1712

The *E. coli* Rosetta strain was transformed with the expression vector and grown in 1 L of Luria broth at 37°C until an optical density of 0.6 was reached. Then 0.5 mM isopropyl-β-d-thiogalactopyranoside was added to the culture, which was maintained at 37°C for 3 h. The cells were harvested by centrifugation and resuspended in a total volume of 40 ml of buffer A (50 mM Tris, 1 M NaCl, pH 8.0) and lyzed by sonication for 20 min at 4°C using a Branson 250 sonicator (duty cycle 80%, output control 4). After a centrifugation (20 000 × *g*for 30 min at 4°C), the supernatant was submitted to further purification. The sample was loaded on a Chelating Sepharose Fast Flow column (1 × 30 cm, GE Healthcare) charged with Ni^2+^ previously equilibrated with buffer A. After washing the column with buffer A supplemented with imidazole 10 mM, an imidazole gradient was applied to elute the recombinant TTC1712 protein (10 to 500 mM, 10 column volumes). Pooled fractions containing TTC1712 were concentrated by ultrafiltration (Amicon, 10 kDa cut-off, Millipore Merck) and further purified by gel filtration chromatography using a Superose 12 10/300 GL column (GE Healthcare) in buffer (50 mM Tris, 1 M NaCl, glycerol 5%, pH 8.0). The fractions of interest were dialyzed against buffer (50 mM Tris, 100 mM NaCl, 5% glycerol, pH 8.0) and concentrated by ultrafiltration to reach a concentration of 10 mg/ml. The purity of the protein was assessed by sodium dodecyl sulfate–polyacrylamide gel electrophoresis with Coomassie blue staining and shown to be above 95%. The sample was then frozen in liquid nitrogen and stored at −80°C until use. TTC1712 variants were purified using Ni-TED Protino columns (Macherey-Nagel) with the same buffer as described above.

### Preparation of *T. thermophilus* rRNA

*Thermus thermophilus* cells were grown to late exponential phase at 70°C in 1 L of Tryptic Soy Broth (TSB). Cells were harvested by centrifugation and the pellet was resuspended in 20 ml buffer B (50 mM Tris–HCl, 10 mM MgCl2, pH 8.0). The cells were disrupted by sonication for 20 min at 4°C using a Branson 250 sonicator (duty cycle 80%, output control 3). The lysate was cleared by centrifugation (12 000 × *g* for 30 min). Ribosomes were precipitated using 1 volume of buffer (20% w/v PEG4000, 50 mM Tris–HCl, 10 mM MgCl_2_, pH 8). After centrifugation (12 000 × *g* for 30 min at 4°C), the pellet was resuspended in 20 ml buffer B and the rRNAs were extracted by non-buffered phenol, and the rRNAs were precipitated with 1/10 volume of AcONa 3 M pH 6.5 and an equal volume of isopropanol. The pellet was dissolved in H_2_O.

### Preparation of *T. thermophilus* tRNA

*Thermus thermophilus* cells were grown to late exponential phase at 70°C in 1 L of TSB. Cells were harvested and the pellet was resuspended in H_2_O. Then an equal volume of buffer (100 mM NaOAc, 20 mM MgCl_2_, 300 mM NaCl, pH 4.5) was added. After two extractions with non-buffered phenol, nucleic acids were precipitated with 1/10 volume of 20% KOAc pH 4.5 and an equal volume of isopropanol. The pellet was dissolved in buffer C (30 mM NaOAc, 10 mM MgCl_2_, pH 5.5) and applied to a DEAE Sepharose Fast Flow column (1 × 5 cm, GE Healthcare) equilibrated with buffer C. The column was washed with the same buffer supplemented with 400 mM NaCl, and tRNAs were eluted with a linear gradient of NaCl (400 to 800 mM, 10 column volumes). The fractions containing the tRNAs were pooled. These fractions also contain the 5S rRNA.

### Ribosomal profile analysis by sucrose density gradient centrifugation

The analysis of ribosomal profiles was performed by sucrose density gradient (SDG) centrifugation [22, 24]. *Escherichia coli* and *T. thermophilus* cells were cultivated in, respectively, 250 ml of LB at 30°C or 250 ml of TSB at 70°C, until A_660_ reached 0.5, chilled at 4°C for 2 h and harvested by centrifugation. The cells were suspended in 3 ml of buffer D1 [20 mM HEPES–KOH, 0.5 mM Mg(OAc)_2_, 200 mM NH_4_Cl, 6 mM β-mercaptoethanol, pH 7.6] or D2 [20 mM HEPES–KOH, 10 mM Mg(OAc)_2_, 30 mM NH_4_Cl, 6 mM β-mercaptoethanol, pH 7.6]. Cells were sonicated for 20 min at 4°C using a Branson 250 sonicator (duty cycle 80%, output control 2). The lysate was clarified by centrifugation (16 000 × *g* for 30 min at 4°C) and 7.5 A_260_ units were layered on top of a sucrose gradient [10%–35% (w/v)] in buffer D1 or D2 supplemented with 13 U/μl heparin (except for samples dedicated to MTase enzymatic assays, for which heparin was omitted) and separated by ultracentrifugation in a Beckman SW-41Ti Rotor at 37 000 rpm for 5 h at 4°C. Ribosomal subunits were fractionated on a Piston Gradient Fractionator and the A_260_ was measured using a UV monitor.

### Preparation of ribosomal subunits

Ribosome subunits were purified by SDG centrifugation as described above and precipitated using 400 μl of buffer (20% w/v PEG4000, 50 mM Tris–HCl, 10 mM MgCl_2_, pH 8). Samples were incubated at room temperature for 10 min. After centrifugation (12 000 × *g* for 30 min at 4°C), ribosomal subunits were resuspended in 9 μl of buffer D1 or D2.

### Extraction of 16S and 23S rRNA from ribosomal subunits

Ribosome subunits were purified by SDG centrifugation as described above. The 16S and 23S rRNA were extracted from the 30S and 50S fractions, according to the instructions of the TRIzol Reagent kit (Invitrogen).

### RNA methyltransferase assays

A semi-quantitative method to follow RNA methylation *in vitro* consisted of measuring the amount of radioactivity transferred to RNA isolated from bacterial cells (10 μg), to rRNA transcripts (2 μg) or to synthetic RNA (quantities are indicated in the legend of figures) using [methyl-^3^H or ^14^C]-SAM as the methyl donor. The reaction mixture (200 μl) consisted of the RNA, the enzyme and 1 μCi [methyl-^3^H]-SAM (82 Ci/mmol) or 20 nCi [methyl-^14^C]-SAM (58 mCi/mmol) in the reaction buffer (50 mM Tris–HCl, 5 mM MgCl_2_, pH 8.0). 2 μg, 1 μg, or 0.1 μg TTC1712 were used to methylate RNA isolated from bacterial cells, synthetic RNA or, rRNA transcripts, respectively. Unless otherwise specified in the legend, the mixture was incubated at 50°C for 30 min or 60 min in the case of reactions intended for two-dimensional thin-layer chromatography (2D-TLC). The reaction was stopped by phenol extraction and the nucleic acids were TCA-precipitated. Radioactive methylated RNA was captured on a Whatman GF/C filter and washed three times with ethanol prior to the measurement of radioactivity in a scintillation counter.

For modified nucleotide identification, the RNA methylated in the presence of [methyl-^14^C]-SAM was ethanol precipitated after phenol extraction and thereafter hydrolysed by nuclease P1. Modified nucleotides were analysed by 2D-TLC on cellulose plates (Merck). The first dimension was developed with solvent A (isobutyric acid/concentrated NH_4_OH/water; 66/1/33; v/v/v); the second dimension was developed with solvent B [0.1 M sodium phosphate at pH 6.8/solid (NH_4_)_2_SO_4_/n-propanol; 100/60/2; v/w/v] or solvent C (concentrated HCl/2-propanol/water; 17.6/68/14.4; v/v/v). The migration pattern was visualized by autoradiography. The nucleotides were identified using a reference map [34].

### Minimal inhibitory concentration determination

To determine the minimal inhibitory concentration (MIC) of antibiotics for *T. thermophilus* wild-type (WT) and Δ*ttc1712* strains, 50 μl of exponentially grown cultures were inoculated in 3 ml of TSB supplemented with different concentrations of antibiotic and incubated overnight at 60°C. The minimal antibiotic concentration that completely inhibited growth was defined as MIC.

### Electrophoresis mobility shift assay

The U2552 59-mer RNA (100 pmol) was annealed for 5 min at 75°C in a binding buffer (100 mM MOPS, 5 mM MgCl_2_, 250 mM KCl, pH 7.0). The protein–RNA complexes were prepared by adding the enzyme (220 pmol, unless otherwise indicated) and incubating for 10 min at 70°C in a total reaction volume of 6 μl. When present, SAM and *S*-adenosyl-homocysteine (SAH) were added at a concentration of 5 mM prior to incubation. For each sample, free RNA and complexes were separated on a 6% acrylamide native gel run for 90 min at 120 V and visualized by ethidium bromide staining.

### Mass spectrometry

#### Isolation of a specific RNA fragment

For LC–MS/MS mass spectrometry analysis, a 57 nt fragment of *T. thermophilus* 23S rRNA containing U2552 (from 2515 to 2571) was obtained by RNase H (Thermo Fisher Scientific) cleavage of RNA regions complementary to the DNA oligonucleotides MS-1 and MS-2 [35]. The 23S rRNA digestion was performed in RNase H buffer (20 mM Tris–HCl, 40 mM KCl, 8mM MgCl_2_, 1 mM dithiothreitol (DTT), pH 7.8) for 2 min at 80°C followed by slow cooling to 50°C and incubation with 0.5 U of RNase H for 20 min at 50°C. The RNase H fragment was isolated by denaturing (8 M urea) 10% polyacrylamide gel electrophoresis. The corresponding band was excised under UV light for LC–MS/MS analysis.

#### Mass spectrometry analysis of the isolated fragment

LC–MS/MS analysis was done as already described [36]. Briefly, gel pieces containing the RNase H fragment previously isolated were digested by 20 μl of 0.1 U/μl RNase T1 (Thermo Fisher Scientific) during 4 h at 50°C. Samples were desalted using ZipTip C18 (Millipore) by several washes with 200 mM ammonium acetate and elution with 50% acetonitrile in milli-Q water and finally dried under vacuum. The pellet containing RNase digestion products was resuspended in 3 μl of milli-Q water. The products were separated on an Acquity peptide BEH C18 column (130 Å, 1.7 μm, 75 μm × 200 mm) using a nanoAcquity system (Waters). The column was equilibrated in a buffer containing 7.5 mM triethylammonium acetate, 7.0 mM triethylamine, and 200 mM hexafluoroisopropanol at a flow rate of 300 nl/min. The column was achieved using a gradient from 15% to 35% methanol for 2 min and the oligonucleotides were eluted with an increase of methanol up to 50% in 20 min. MS and MS/MS analysis was performed using a SYNAPT G2-S instrument (Waters). All experiments were performed in negative mode with a capillary voltage set at 2.6 kV and a sample cone voltage set at 30 V. The source was heated to 130°C. Samples were analysed over an m/z range from 500 to 1500 for the full scan, followed by a fast data direct acquisition scan (Fast DDA). Collision-induced dissociation (CID) spectra were deconvoluted using MassLynx software (Waters) and manually sequenced by following the y and/or c series.

### Crystallization, data collection, and structural characterization

Crystallogenesis was performed at 20°C by the under-oil crystallization procedure, using 18 μl paraffin oil to cover the crystallization drops. Crystals of TTC1712 apoenzyme were obtained by mixing 1 μl of pure TTC1712 (10 mg/ml) with 1 μl of crystallization solution (100 mM Na citrate, pH 4, 20% PEG6000, 1 M LiCl). Crystals of TTC1712-RNA or TTC1712-RNA in the presence of SAH (TTC1712-RNA-SAH) or SAM (TTC1712-RNA-SAM) were obtained as follows. Five microlitres of TTC1712 (10 mg/ml) were incubated with 2.65 μl of RNA (U2552 59-mer RNA, 500 μM), first preincubated at 70°C for 2 min. The protein–RNA sample was further incubated for 2 min at 70°C and slowly cooled down to 4°C. A volume of 0.18 μl of SAH or SAM (50 mM) was added just prior to crystallogenesis. Crystals were obtained following the same method as for the apoenzyme, with crystallization solution (100 mM Na citrate, pH 4.5, 10% PEG6000, 1 M LiCl). Streakseeding was used to initiate the crystallization, using crystals of the apoenzyme. Crystals typically grew within a week and were analysed at the SOLEIL Proxima 1 and 2 beamlines. The XDS and AutoPROC [37, 38] packages were used for data processing. Phenix was used for model building and refinement [39]. The structure was solved by molecular replacement, with TTHA0275 (pdb code: 4X3M) as search model. The RNA was manually built in the electron density.

Data collection and refinement statistics are presented in Supplementary Table S2. PyMOL was used as a molecular graphic tool to prepare the figures (The PyMOL Molecular Graphics System, Version 2.0 Schrödinger, LLC). The PDBSum software was used to create a 2D topology diagram [40].

## Results

### TTC1712 catalyzes Um formation at position 2552 of 23S rRNA of *T. thermophilus*

In the context for the search of the enzyme forming Um2552 in the 23S rRNA of *T. thermophilus*, it is worth mentioning that *Bacillus subtilis* does not contain Um2552 but Gm2553, a 2′-*O*-methylation at position adjacent to U2552 in 23S rRNA. The enzyme RlmP catalysing this methylation has recently been identified [35]. RlmP belongs to class IV of MTases and contains an L30-like N-terminal RNA-binding domain and a catalytic SPOUT C-terminal domain. Hence, we searched for homologs of RlmP in *T. thermophilus*, considering that one of these proteins could form Um2552.

A BLAST search with RlmP as the query, revealed several *T. thermophilus* ORFs among which *ttc1712*, *ttc1691*, and *ttc1867* showed the best scores (124, 87, and 63.5, respectively). *ttc1691*and*ttc1867* encode RlmB and TrmH, the enzymes forming, respectively, Gm2251 in 23S rRNA and Gm18 in tRNA, whereas *ttc1712* encodes a putative rRNA MTase. This prompted us to consider the product of ORF *ttc1712* as a candidate for Um2552 formation in *T. thermophilus*. Whether TTC1712 is responsible for Um2552 formation was determined by two complementary approaches: inactivation of the corresponding gene by the insertion of a kanamycin resistant cassette [31] and *in vitro* testing of the purified recombinant TTC1712 enzyme activity.

**Figure 1.**
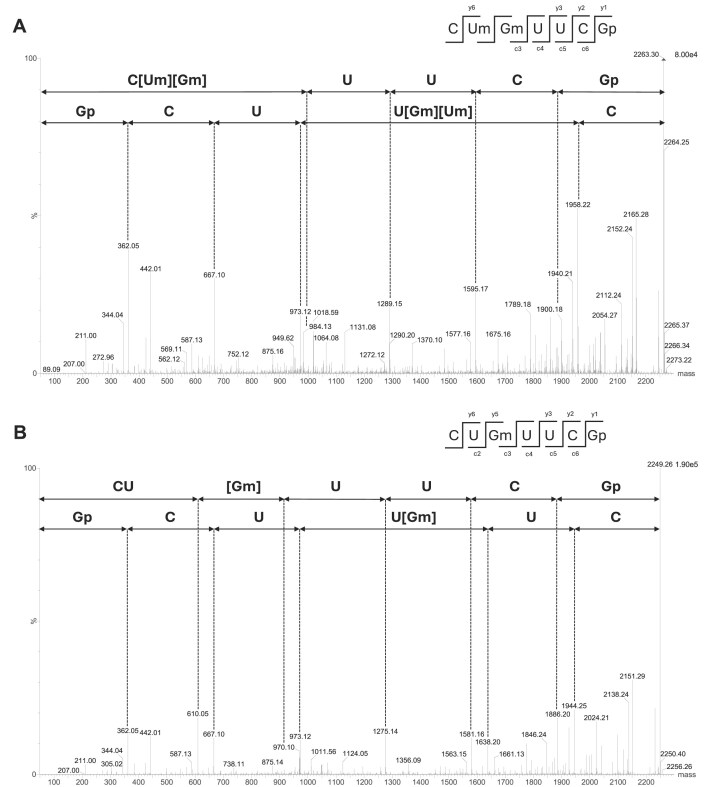
Localization of the TTC1712 target nucleoside through mass-spectrometry analysis. The RNA portions corresponding to the A-loop of *T. thermophilus* WT and *Δttc1712* 23S rRNA, obtained by T1 digestion of an RNase H 2515–2571 fragment, were compared. To obtain a fragment of an appropriate length amenable to MS/MS analysis, extracted rRNA was previously *in vitro* 2′-*O*-methylated at position G2553 by RlmP. (**A**) MS/MS sequencing spectrum of 2263.3 Da (m/z 1131.1, *z*= 2−) sequence C[Um][Gm]UUCGp, obtained from the analysis of the RNA fragment from the WT strain. CID fragmentation shows methylation of U2552 and G2553. (**B**) MS/MS sequencing spectrum of 2249.3 Da (m/z 1124.13, *z* = 2−) sequence CU[Gm]UUCGp, obtained from the analysis of the RNA fragment from the *Δttc1712* strain. CID fragmentation shows the absence of methylation of U2552.

The modification status of U2552 in the 23S rRNA of the *ttc1712* deletion strain was determined by mass spectrometry. LC MS/MS analysis of rRNA from WT cells revealed the presence of the sequence C[Um][Gm]UUCGp (mass of 2263.3 Da) and MS/MS fragmentation confirmed methylation at position 2552 (Fig. 1), whereas the sequence CU[Gm]UUCGp (mass of 2249.3 Da) was found with the rRNA isolated from the *Δttc1712* mutant. Notably, no ion corresponding to UGm was detected in the RNA extracted from the WT strain, indicating that U2552 is fully modified *in vivo*. The modification Um2552 is thus present in the WT strain and absent in the mutant. This result is consistent with TTC1712 targeting uridine 2552 of 23S rRNA of *T. thermophilus in vivo*.

**Figure 2.**
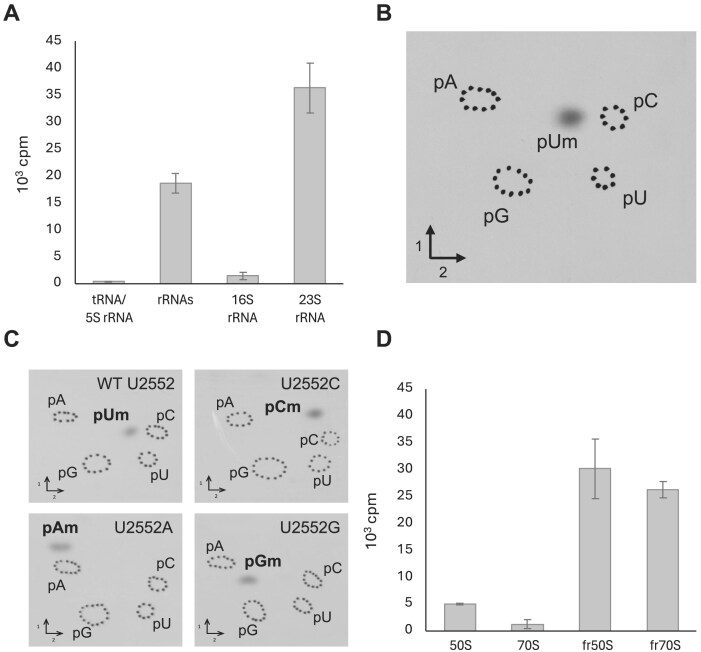
(**A**) TTC1712 specifically modifies the 23S rRNA *in vitro*. tRNA and rRNAs from *T. thermophilus Δttc1712* were tested as TTC1712 substrates. Values represent the mean (*n* = 2), with the error indicated as half of the absolute difference between the replicates. (**B**) TTC1712 catalyzes the formation of Um in *T. thermophilus* rRNA. Autoradiography of 2D chromatograms of P1 hydrolysates of *T. thermophilus Δttc1712* rRNA, methylated by TTC1712 *in vitro*. The nucleotides were identified using reference maps [34]. (**C**) TTC1712 methylates the ribose of nucleotides C, A, and G when U2552 is mutated in 23S rRNA transcripts. Autoradiography of 2D chromatograms of P1 hydrolysates of these *in vitro* transcripts, methylated by TTC1712. The nucleotides were identified using reference maps [34]. (**D**) Fully assembled 70S ribosome and 50S large subunit are poor *in vitro* substrates of TTC1712. 70S or 50S particles from *T. thermophilus Δttc1712* were incubated with TTC1712 and [methyl-^3^H]-SAM. rRNA-fr50S or rRNA-fr70S are for rRNA extracted from 50S or 70S particles, respectively. Values represent the mean (*n* = 2), with the error indicated as half of the absolute difference between the replicates.

In the second approach, the recombinant TTC1712 protein was purified and tested with various RNA substrates. rRNA and tRNA were extracted from the *T. thermophilus* Δ*ttc1712* mutant and tested as TTC1712 substrates. Note that the rRNA preparation contains 16S, 23S and 5S rRNAs, with the latter also present in the tRNA preparation. The results show that TTC1712 methylates 16S and 23S rRNA, but not 5S rRNA or tRNA (Fig. 2A). To identify the specific rRNA modified, 16S and 23S rRNAs were extracted from the 30S and 50S subunits of the mutant strain, respectively, and tested as substrates. The 23S rRNA was modified, whereas 16S rRNA was not. To determine the nature of the modification, rRNA was methylated *in vitro* by TTC1712, then hydrolysed and analysed by 2D-TLC. The results (Fig. 2B) show the formation of a radioactive nucleotide matching the characteristics of pUm [34].

Finally, to identify the exact position modified by TTC1712, we also performed this assay using a WT T7 transcript corresponding to *T. thermophilus* 23S rRNA (U2552), and T7 transcripts with U2552 mutated to C, A, or G. The results presented in Fig. 2C show the formation of Um, Cm, Am, or Gm in these transcripts. This indicates that the enzyme modifies the 2′-O of the nucleoside at position 2552 and is independent of the nature of the base.

Taken together, these results demonstrate that the protein encoded by ORF *ttc1712* is the 23S rRNA MTase responsible for the 2′-*O*-methylation of U2552 in *T. thermophilus*. According to the bacterial nomenclature of rRNA MTases, we therefore renamed protein TTC1712 as RlmR.

### Loss of RlmR does not result in a detectable phenotype as observed in *E. coli*

To determine whether growth is affected by the loss of RlmR, the growth rates of WT and mutant *ΔrlmR T. thermophilus* strains were compared. At 70°C, the doubling times for both strains were similar (WT: 71 ± 7 min; *ΔrlmR*: 68 ± 12 min). Next, since modification of rRNA is known to modulate the sensitivity to several antibiotics, we studied the effect of various antibiotics targeting the peptidyl transferase centre in the 50S subunit. However, the lack of U2552 methylation did not change the resistance to chloramphenicol, linezolid, lincomycin, and puromycin (Supplementary Table S3). Additionally, to assess the potential involvement of RlmR in *T. thermophilus* resistance to thermal stress, cells were submitted to various temperature shocks, ranging from 15 to 95°C, and viability was tested at 70°C on solid medium. No difference was observed under these conditions (Supplementary Fig. S1).

**Figure 3.**
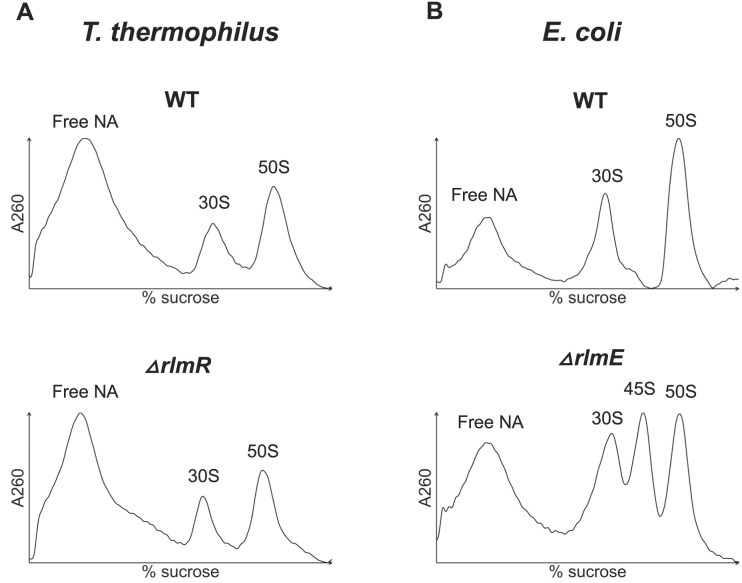
SDG profile of ribosomal subunits performed at low magnesium concentration from (**A**) *T. thermophilus* WT and *ΔrlmR* grown at 70°C and (**B**) *E. coli* WT and *ΔrlmE* grown at 30°C.

Finally, the role of RlmR on ribosome biogenesis was investigated. First, to assess whether RlmR is a late actor in ribogenesis or plays a role at an earlier step between transcription and the final stages of mature 50S subunit formation, 50S and 70S particles from the mutant *ΔrlmR* strain were tested as substrates for RlmR *in vitro*. These particles were purified by ultracentrifugation in SDG. Figure 2D shows that RlmR is not able to methylate the 70S ribosome or the 50S subunit, whereas deproteinized rRNA, extracted from these ribosomal particles, is efficiently modified *in vitro*. Additionally, since the loss of RlmE in *E. coli* is known to cause an accumulation of a 45S ribosomal intermediate, we investigated the potential impact of RlmR absence on ribosome biogenesis using SDG analysis (Fig. 3 and Supplementary Fig. S2). The results presented in Fig. 3 show no alteration in ribosome profile of the *ΔrlmR* strain compared to *T. thermophilus* WT, whereas the profile of the *E. coli ΔrlmE* was significantly affected.

### Structure of RlmR apoenzyme

A search in the Protein Data Bank for RlmR orthologs revealed the 3D structure of TTHA0275 from *T. thermophilus* HB8 (pdb codes 4X3M and 4X3L; unpublished result). However, the deposited structures contain either methylthioadenosine (MTA) or adenosine (ADN), two SAM degradation products, bound to the active site of the enzyme. Therefore, the study of the structure of the *T. thermophilus* HB27 enzyme in the absence of any SAM derivative was undertaken. The structure of the enzyme from HB27 and HB8 differs by only three amino acids, and apart from the slightly different orientation of the L30-like domain (see below), both proteins are identical (Root mean square deviations are 0.690 and 1.472 Å for TTHA0275-MTA and TTHA0275-ADN, respectively).

**Figure 4.**
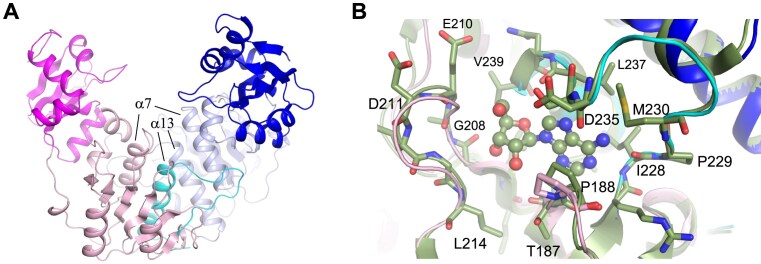
3D structure of RlmR. (**A**) Overall structure of the RlmR dimer, represented as cartoon. The subunits A and B are coloured in magenta and blue, respectively, with the N-terminal domain in a darker colour and residues 226–260, which participate in the knot formation, in cyan. (**B**) Overlay of RlmR [coloured as in panel (A)] with TTHA0275-ADN (green). The ADN is represented as sticks and spheres. The side chain of the main residues of the cofactor binding site are represented as sticks.

The RlmR overall structure consists of N-terminal (1–96) and C-terminal (∼105–260) domains (NTD and CTD) joined by a linker. The enzyme crystallizes as a dimer, the asymmetric unit consisting of two dimers. Dimerization involves around 1535 Å^2^ interface consisting of CTD α-helices α7 and α13 of both protomers, organized perpendicularly (Fig. 4A and Supplementary Fig. S3). The dimeric state, confirmed by gel filtration chromatography (Supplementary Fig. S4), and the perpendicular interface organization observed for RlmR are hallmarks of SPOUT enzymes acting on the ribose, in contrast to the parallel dimerization mode adopted by SPOUT enzymes acting on the base [7]. To date, only Sfm1 and Trm10 were shown to function as monomers [41, 42]. The CTD of RlmR adopts the classical SPOUT MTase fold, with a central core consisting of a β-sheet made of seven parallel β-strands sandwiched between seven α-helices [43]. The CTD contains the three sequence motifs (Supplementary Fig. S5) and a deep trefoil knot, which are both characteristic of the SPOUT superfamily [44, 45]. The topological knot participates in the formation of the SAM binding site and stabilizes the adenine moiety of SAM, which interacts with residues P188, I228, M230, and L237 (Fig. 4B). Except for a slight movement of P188 side chain to allow a CH-π interaction with the adenine, no major active site difference was observed between the *T. thermophilus* HB27 apoenzyme and TTHA0275 in complex with MTA or ADN.

The NTD of RlmR shows high structural similarity with the ribosomal proteins L7Ae/L30 (eL8/eL30 in the new nomenclature [46]) and is suggested to function as the RNA binding domain [47–49]. Among the SPOUT proteins of known function, RlmR shares high structural and sequence similarities with RlmP (or MRM3), RlmB and several MTases conferring resistance to various antibiotics (Table 1).

**Table 1.** RlmR structural homologs of known functions

	Source	Modification (*E. coli* numbering)	% identity	Pdb code	Reference
RlmR	*Thermus thermophilus*HB27	Um2552	100	9MUK	This study
RlmR	*Thermus thermophilus*HB8	Um2552	98.9	4X3L	Unpublished
RlmP	*Bacillus subtilis*	Gm2553	32.6	7QIU	[35]
MRM3	*Homo sapiens*	Gm2553	30.5	7OI6	[57]
RlmB	*Escherichia coli*	Gm2251	28.7	1GZ0	[73]
AviRB	*Streptomyces viridochromogenes*	Um2479	30.9	1X7P	[74]
Tsr	*Streptomyces cyaneus*	Am1067	23.9	3GYQ	[75]
NHR	*Streptomyces actuosus*	Am1067	23.8	3NK6	[76]

### Structure of RlmR in complex with a substrate RNA

To date, there is no high-resolution structure of a SPOUT 2′-*O*-MTase in complex with its substrate RNA, which prevents the in-depth understanding of the molecular determinants allowing specific RNA recognition. Moreover, the catalytic mechanism of SPOUT 2′-*O*-MTases remains hypothetical and mainly based on mutational and *in silico* studies [50–53].

**Figure 5.**
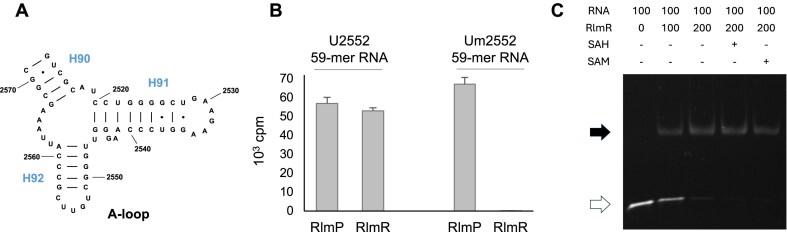
(**A**) Secondary structure of U2552 59-mer RNA, comprising residues 2513–2571 of *T. thermophilus* 23S rRNA, corresponding to H90 to H92. (**B**) Activity of *T. thermophilus* RlmR on 59-mer synthetic RNAs used in this study. Twenty micrograms of RNAs were incubated with 1 μg RlmR and [methyl-^3^H]-SAM during 90 min. *Bacillus subtilis* RlmP was used as a control to confirm the integrity of the synthetic RNAs. The values are the mean ± standard deviation (*n* = 3). (**C**) RlmR binds RNA as a 2:1 complex. Electrophoretic mobility shift assay with U2552 59-mer RNA in the presence of RlmR, with both quantities expressed in pmol. The position of free RNA and RlmR-RNA complex is indicated with a white or black arrow, respectively. When present, SAH or SAM were added at a concentration of 5 mM.

We therefore determined the crystal structure of RlmR in complex with a synthetic RNA. To this end, a 59-mer RNA corresponding to the *T. thermophilus* 23S rRNA A-loop, centered on U2552, was designed (U2552 59-mer RNA). To maximize the rigidity of the RNA fragment and thus optimize the chances of crystallization, the A-loop was extended to helices H92 and H91, as well as a part of H90 (Fig. 5A).

We first verified, using an *in vitro* MTase activity assay, that the designed RNA fragment could serve as a substrate for RlmR. A high activity was indeed observed, which was abolished when an RNA fragment already 2′-*O*-methylated at position 2552 was used (Fig. 5B). Noteworthy, *B. subtilis* RlmP was used as a control to confirm the integrity of the 2′-*O*-methylated RNA. These results confirmed the specificity of the enzyme for U2552 on this synthetic substrate. Moreover, an electrophoretic mobility shift assay (EMSA) experiment confirmed that RlmR forms a 2:1 complex with the unmodified 59-mer RNA fragment, which is in line with the fact that SPOUT enzymes are usually active as dimers (Fig. 5C). The presence of SAM or SAH does not affect the formation of the RlmR-RNA complex. Taken together, these results show that RlmR does recognize an RNA fragment of limited size and that long-range interactions with distant portions of the RNA are not mandatory for RNA methylation.

**Figure 6.**
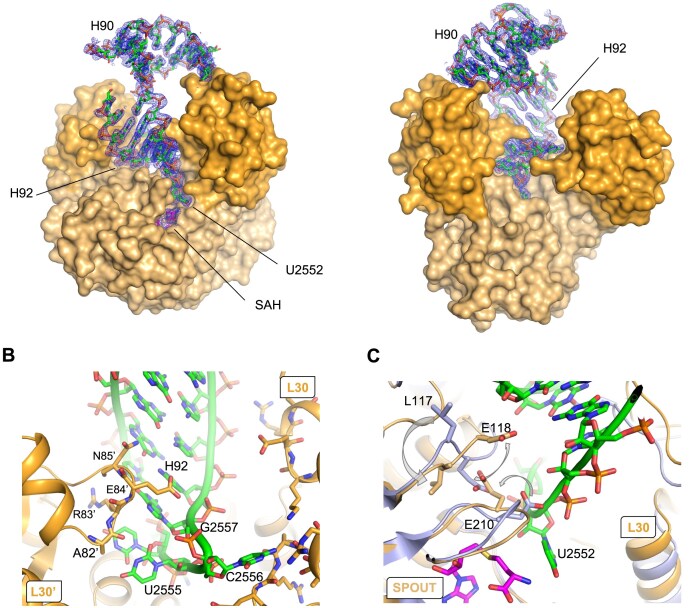
Crystal structure of RlmR in complex with the U2552 59-mer synthetic RNA and SAH. (**A**) Front and back view of the complex with the 2mFo-DFmodel electron density map around the RNA (1*σ* contour). The protein is represented as van der Waals surface with the SPOUT and L30-like domains coloured in light and dark orange, respectively. RNA and SAH are represented as sticks with carbon atoms coloured in green and magenta, respectively. (**B**) View of the L30-like domain (particularly residues 82–85) trapping the RNA through interactions with the A-loop and H92. The protein and the RNA are coloured as in panel (A). (**C**) Overlay of the apoenzyme (light blue) with the RlmR-RNA-SAH complex [coloured as in panel (A)]. The concerted movement of E210, E118, and L117 side chains observed upon RNA binding is depicted by arrows. The single quote symbol refers to protomer B.

To prevent the methylation of the RNA and the dissociation of the complex, first crystallogenesis experiments were performed with RlmR and RNA in the absence of co-substrate. Unexpectedly, crystals were obtained in the same condition as for the apoenzyme. However, only a poor electron density was observed for the RNA in this condition, preventing the building of a model. We next attempted the crystallization in the presence of SAH. This allowed to solve the crystal structure of RlmR in complex with the 59-mer RNA and SAH to a resolution of 1.9 Å. SAH was found in its bent conformation [7]. A clear electron density was observed for the A-loop, H90 and H92 of the RNA (Fig. 6A). In contrast, the stem–loop comprising H91 and nucleotides 2564–2566 of the loop joining H91 and H92 are largely absent from the model, due to the poor electron density. However, based on the position of its first nucleotides, H91 seems to take a different trajectory compared to the corresponding structure in the native *T. thermophilus* 70S ribosome (Supplementary Fig. S7), likely due to the crystal packing [30]. Noteworthy, we also obtained a model of RlmR in complex with U2552 59-mer RNA from the AlphaFold 3 server [54]. In this model, H91 orientation is more similar to that found in the native *T. thermophilus* ribosome. However, it fails to correctly position Um2552 in the active site and to predict the A-loop conformational rearrangement described below. Due to these uncertainties about the correct positioning of H91, and the potential impact of H91 positioning on H90 conformation, the protein–RNA interactions involving these RNA regions will not be discussed in this article.

### RlmR dynamics

In the absence of RNA, the structures of RlmR apoenzyme (this study), TTHA0275-MTA and TTHA0275-ADN show several different orientations of L30-like domains relative to the SPOUT domains (Supplementary Fig. S8A). This reflects an intrinsic interdomain dynamic, which is also predicted based on a normal mode analysis performed on RlmR (Supplementary Fig. S8B). Following RNA binding, a significant inward pivotal motion of the L30-like domains occurs and leads to a narrowing of the groove intended to accommodate the RNA. To illustrate this conformational change, we measured the E19-E84’ distance, which limits the opening created by both domains (the single quote symbol refers to protomer B throughout this article). This distance decreases from ∼17 to ∼10 Å upon RNA binding (Supplementary Fig. S8C). This motion brings the L30-like domains, including residues 78–84 and 18–21 of protomer A, closer to the RNA, thus allowing interactions with the substrate. Moreover, the loop encompassing L30-like domain residues A82′ to N85′ covers part of H92 helix and A-loop, which traps the RNA in its final position (Fig. 6B).

In the heart of the active site, RNA binding is accompanied by conformational changes in loops 210–212 and 117–120 (Fig. 6C). While the loop 210–212 is poorly defined in the apoenzyme, it is stabilized in the functional active site following RNA binding. Moreover, U2552 nucleotide triggers a backward movement of E210 side chain, avoiding a steric clash. In a cascade event, E118 and L117 residues are flipped.

### RNA dynamics

**Figure 7.**
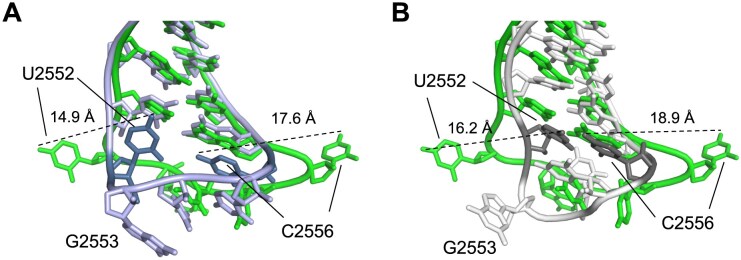
Comparison of the A-loop conformation in the RlmR-RNA-SAH complex (green) with (**A**) the A-loop conformation in the 70S ribosome of *T. thermophilus* (blue) [30] or (**B**) the NMR structure of a 19-mer RNA comprising U2552 (white) [56]. U2552 and C2556 in the ribosome or the NMR structure are indicated. The distances between N4 atoms of C2556 and O4 atoms of U2552 in the compared conformations are indicated, revealing the drastic conformational change occurring in the A-loop upon RlmR binding.

The conformation of the A-loop in the RlmR-RNA-SAH complex and that in the *T. thermophilus* 70S ribosome were compared [30]. The interaction of RlmR with the RNA triggers a pivotal movement of the A-loop around an axis formed by nucleotides 2551 and 2557 (Fig. 7A). This movement results from a rotation of the phosphodiester bonds between nucleotides 2555–2557 and nucleotides 2551–2553. Moreover, the formation of the complex leads to a different orientation of U2552 and C2556 compared to the 70S ribosome structure. Indeed, in the latter, the bases of these nucleotides point towards the interior of the A-loop. In contrast, they are twisted towards the outside of the loop in the RlmR-RNA-SAH complex. This new conformation of U2552 and C2556 is stabilized by several interactions with the protein, as discussed below. Noteworthy, the orientation of U2552 and C2556 in the A-loop of *T. thermophilus* ribosome is similar to that found in *E. coli* WT or *ΔrlmE* 50S subunits or in the Nuclear Magnetic Resonance (NMR) structure of a 19-mer RNA comprising U2552 [23, 55, 56]. In the NMR solution structure of the A-loop, U2552 and C2556 are further stabilized by the formation of a non-canonical base pair (Fig. 7B). Therefore, the distinct conformation observed for these nucleotides in the RlmR-RNA-SAH complex cannot be attributed to the methylation status of U2552 or to the overall ribosomal context of the A-loop. Instead, these structural data provide strong evidence that the observed conformational flip represents a genuine induced fit resulting from binding to RlmR.

**Figure 8.**
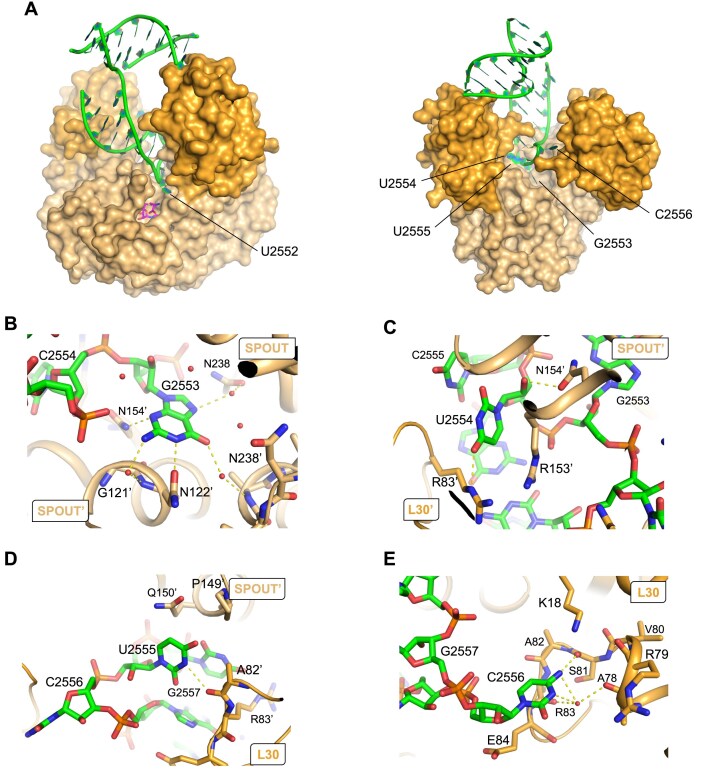
Interactions of RlmR with nucleotides 2553–2556 from the RNA A-loop. The RlmR SPOUT and L30-like domains are coloured in light and dark orange, respectively. RNA and SAH are coloured in green and magenta, respectively. (**A**) Front and rear views of the RlmR-RNA-SAH complex. (**B**–**E**) Focus on nucleotides G2553 to C2556. Selected RlmR residues interacting with the RNA are highlighted as sticks and labelled.

The hinge motion and the associated flip of nucleotides 2552 and 2556 contribute to the complete remodelling of the A-loop, which leads to several consequences. It prevents steric clashes that would otherwise occur between RlmR and the A-loop. In the herein described crystal structure, the A-loop fits optimally in the central groove formed at the interface between the two protomers and creates extensive Van der Waals contacts with the enzyme (Fig. 8A and Supplementary Fig. S6). Among others, G2553 interacts with G121′ and N122′ (motif I), N154′ and several water molecules (Fig. 8B). O4 and O2′ atoms of U2554 interact, respectively, with the R83′ and N154′ side chains (Fig. 8C). Moreover, the N3 atom of U2555 base interacts through a hydrogen bond with A82′ main chain oxygen (Fig. 8D). The base of nucleotide C2556 is stabilized within a pocket bordered by residues 78–84 and K18 (Fig. 8E). Interactions involving U2552 are discussed below.

**Figure 9.**
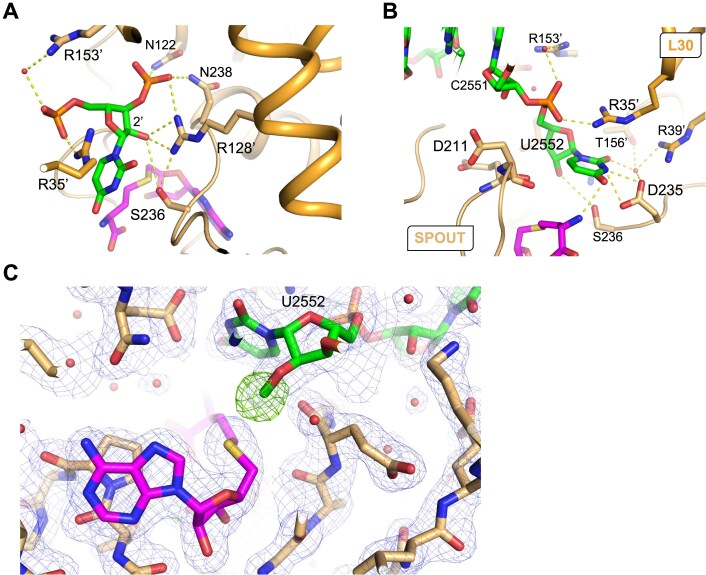
Interactions of RlmR with the target nucleotide U2552. The RlmR SPOUT and L30-like domains are coloured in light and dark orange, respectively. RNA and the co-substrate are coloured in green and magenta, respectively. (**A**, **B**) In the RlmR-RNA-SAH complex, the target nucleotide U2552 is optimally positioned with respect to the catalytic R128′ residue and SAH sulphur atom. Together with N122 and N238, the conserved R128′ interacts with the 3′-phosphate of U2552. S236 is also located at hydrogen bond distance of R128′ and the target ribose O2′. (**C**) Crystal structure of RlmR co-crystalized in the presence of U2552 59-mer synthetic RNA and SAM. The electron density map highlights the transfer of the methyl group from SAM to the 2′-O atom of U2552. The 2mFo-DFmodel electron density map (1.5*σ* contour) around the RNA and the mFo-DFmodel omit electron density map for Um2552 2′-*O*-methyl group in (4*σ* contour) are coloured in blue and green, respectively. The single quote symbol refers to protomer B.

Regarding the target nucleotide, the remodelling positions U2552 optimally within the active site, allowing the methylation of the ribose O2′. As stated above, the U2552–C2556 interaction is disrupted after binding to the enzyme. Extensive polar interactions stabilize the U2552 in its new conformation. Indeed, the U2552 5′-phosphate moiety makes an ionic bond with R35′ and interacts with R153′ side chain through a bridging water molecule. The 3′-phosphate is stabilized by R128′ and interacts with N122 and N238 (motif I and III, respectively) (Fig. 9A and Supplementary Fig. S5). Moreover, the base of U2552 is sandwiched between R35′ guanidino group and S236, which belongs to motif III of SPOUT MTases (Fig. 9B). It also makes polar contacts with D235 carboxyl, SAH amino terminal group and a water molecule stabilized by R39′ and T156′ side chains. Finally, the ribose O2′ is stabilized by S236 Oγ. Most importantly, the target hydroxyl group of U2552 is positioned at 2.8 Å of the catalytic R128′ guanidino group (motif I), a catalytic residue identified in TrmH [53], and at 4.6 Å of SAH sulphur (Fig. 9A). The complex thus reveals the optimal positioning of the target U2552 hydroxyl group with respect to the catalytic R128′ and SAH sulphur atom.

### Mutagenesis of RlmR residues involved in RNA interaction

The structure of RlmR in complex with the RNA reveals a series of RlmR residues involved in RNA interaction. To evaluate their role in the RNA binding, we conducted an EMSA experiment using the U2552 59-mer RNA and several RlmR variants in which these residues were substituted to alanine.

Most mutations had no perceptible effect on RNA binding (Supplementary Fig. S9), presumably due to the broad network of interactions stabilizing the RlmR-RNA complex, which makes a single mutation insufficient to disrupt binding. However, a decrease in RNA binding was observed for N122A, R128A, R153A, N154A, and N238A mutants.

**Figure 10.**
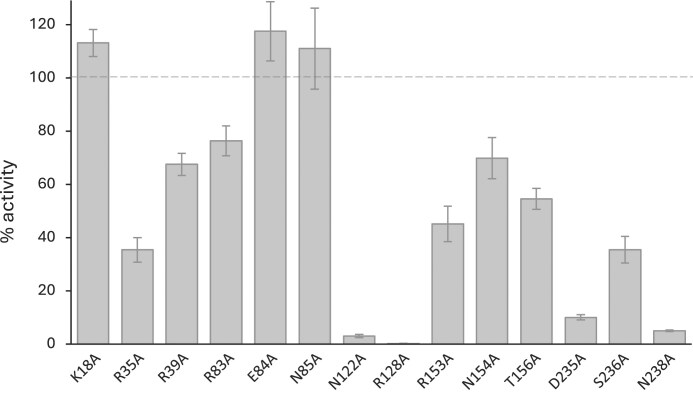
Methyltransferase activity of various RlmR mutants. Residues were selected based on the structure of the RlmR-RNA complex and were substituted to alanine. The assay was performed as described in the methods, using 20 pmol of synthetic U2552 59-mer RNA. Values represent the percentage of activity of the RlmR variants compared to the WT enzyme and are expressed as the mean ± standard deviation (*n* = 4).

To further investigate the contribution of these residues to RlmR activity, we measured the methyltransferase activity of the mutants using U2552 59-mer RNA as a substrate. As shown in Fig. 10, K18A, E84A, and N85A variants are not impacted. In protomers A and B, these residues interact, respectively, with C2556, primarily through their main chain, and with H92 nucleotides. Since H92 is not involved in the A-loop remodelling, the mutation of these residues may therefore not be sufficient to disrupt the overall RlmR enzymatic activity. Except for these variants, the other mutants exhibited reduced catalytic activity. Among these, R35A, R39A, R83A, R153A, N154A, T156A, and S236A displayed a substantially impaired activity (35%–76% residual activity). Notably, the mutation of N122A, R128A, D235A, and N238A caused a nearly complete loss of activity (0%–10% residual activity). These results confirm that most of the tested residues play a role in the overall enzymatic function of RlmR.

### Structure of RlmR-RNA in a post-reaction state

In order to understand how this complex could evolve to a methylated product once in the presence of SAM, the genuine RlmR co-substrate, we co-crystallized the enzyme with the substrate RNA in the presence of SAM instead of SAH. In these conditions, the crystal structure shows a post-reaction state in which the RNA is 2′-*O*-methylated. Indeed, the electron density map clearly reveals the transfer of the methyl group from SAM to the U2552 ribose (Fig. 9C). Consequently, the co-substrate is converted to SAH in the catalytic site. In contrast, in the other catalytic site of the dimer, not hosting the substrate, the co-substrate remains present as SAM.

With this crystal structure, we could evaluate the consequence of the methylation on the conformation of the RNA and the enzyme. However, apart from the methylation per se, no difference between the RlmR-RNA structures obtained in the presence of SAH and SAM was observed.

Noteworthy, we also solved the structure of RlmR co-crystallized with SAH and an analogue of the 59-mer RNA, chemically methylated in 2′-O position of U2552. This led to the same structure as described above, thus showing that the methylation does not impede the initial binding of the RNA to the enzyme.

## Discussion

The presence of Um2552 in the 23S rRNA of *T. thermophilus* has been reported earlier [30]. However, the enzyme responsible for its formation has remained unidentified. Here, we show that this methylation is catalyzed by the SPOUT MTase encoded by the ORF *ttc1712* of *T. thermophilus*, renamed as RlmR.

As several SpoU MTases acting on specific nucleotides of the 23S rRNA, RlmR is a dimer and contains, in addition to the SPOUT catalytic domain, an L30-like RNA binding domain. Whereas RlmR and RlmP (or MRM3) act, respectively, on U2552 and G2553 in the A-loop (*E. coli* numbering), RlmB modifies G2251 in the 23S rRNA P-loop. Moreover, AviRB and Tsr methylate, respectively, U2476 and A1067 (Table 1), highlighting the remarkable adaptability of this domain combination in directing the enzyme on various targets within the rRNA. In the case of RlmR, the *ttc1712* ORF is located close to the *ttc1691* ORF, which encodes for RlmB. We can thus hypothesize that, starting from a duplication of *ttc1691* gene, evolution has diverted the enzyme from its original target on the P-loop to U2552 on the A-loop.

In the present study, the structure of RlmR was obtained with a synthetic 59-mer RNA centered on the A-loop, which constitutes the first high-resolution structure of a SPOUT 2′-*O*-MTase in complex with an RNA substrate. Actually, the structure of SPOUT MTases has been captured twice in their native context using cryo-electron microscopy, either in a human (MRM3) or a *Trypanosoma brucei* (mt-LAF5/6 heterodimer) mitoribosomal LSU assembly intermediate, solved at, respectively, 5.70 Å and 3.90 Å resolution [57, 58]. However, in the case of MRM3, the target A-loop does not adopt a conformation that allows it to extend deep enough in the RNA binding groove. Therefore, the target hydroxyl group oxygen of G2553 ribose is located around 22 Å away from the putative position of the SAM methyl group and is thus not properly positioned to be methylated. In the case of the mt-LAF5/6 heterodimer, whilst the A-loop conformation is rather similar to that observed in our study, the limited resolution did not allow the authors to identify the target nucleotide. Moreover, as in the case of MRM3, none of the ribose 2′-OH of the nucleotides corresponding to U2552 or G2553 is orientated in a way that would allow methylation.

The study of RlmR in complex with an RNA and SAM (or SAH) provides an in-depth understanding of key structural features of the RNA recognition and methylation process, some of which might be generalized to other SpoU MTases. The structure shows the RNA positioned in the central groove of the enzyme, with the A-loop and H92 stabilized by the L30-like and SPOUT domains of both RlmR protomers. Several residues from the SPOUT motifs, among others, stabilize the A-loop. The whole A-loop interacts with the enzyme, highlighting the importance of the RNA sequence downstream of U2552 for RNA binding and correct positioning of the target nucleotide. This likely constitutes a key molecular factor sustaining RlmR selectivity for U2552 in the A-loop rather than other regions of the 23S rRNA. Besides this position specificity, our results indicate that RlmR lacks base specificity, as it can modify position 2552 regardless of the nucleotide. Based on the structural data, it seems achievable to accommodate a cytosine in the binding pocket. However, it is more surprising to fit an ADN or a guanosine, as there appears to be no sufficient space for a double-ring nucleotide. To explain the results obtained with these artificial substrates, we thus suggest that an induced fit mechanism occurs, enabling the recognition and methylation of purines despite the steric hindrance.

**Figure 11.**
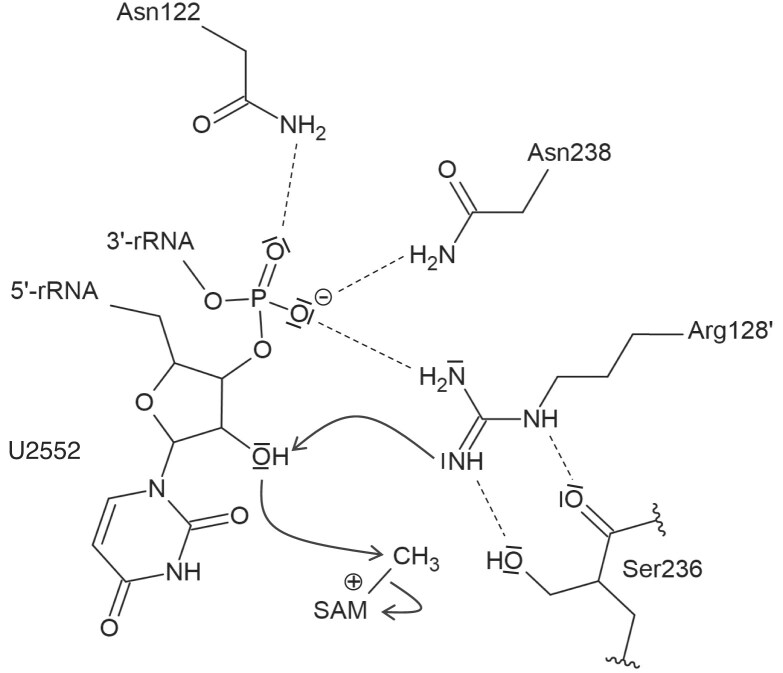
Proposed catalytic mechanism of RlmR from *T. thermophilus*. The U2552 3′-phosphate and Ser236 convert the Arg128′ into the catalytic base. The latter may then activate the ribose 2′-OH, promoting the nucleophilic attack on the SAM methyl group.

To perform the modification, a crucial step resides in the disruption of an internal U2552–C2556 interaction, followed by a flip of both nucleotides. This leads to the complete remodelling of the A-loop. Similar base flipping has been reported for base-modifying SPOUT MTases from the TrmD family, i.e. TrmD, Trm10, and Nep1, and might thus be extrapolated to the whole SPOUT superfamily [7, 59–61]. The described flip exposes the target U2552 and optimally positions its 2′-OH group with respect to the co-substrate and the key catalytic residue R128′. Moreover, the topological knot permits the proper positioning of motif III residues N238 and S236, which are known to be essential for the TrmH-mediated 2′-*O*-methylation of tRNA G18 [52]. Nureki *et al.* proposed a catalytic mechanism for TrmH, based on an *in silico* model, in which the side and main chain oxygen atoms of S150 (S236 in RlmR) as well as the 5′-phosphate of the target nucleotide act together to withdraw a proton from the catalytic arginine R41′ (R128′ in RlmR), thus converting its terminal guanidino group to a catalytic base [53]. This in turn deprotonates and activates the U2552 hydroxyl group, enabling the oxygen atom to perform a nucleophilic attack on the reactive SAM methyl group. Interestingly, while the mutation of S150 leads to a complete loss of activity in TrmH, the corresponding mutation in RlmR results in a variant that still exhibits moderate residual activity, which aligns with the result obtained for Tsr [52, 62]. Whilst our results validate the proposed electron transfer sequence overall, we can assert that the phosphate group involved in the activation of the catalytic arginine is the target nucleotide 3′-phosphate, instead of the 5′-phosphate (Figs 9A and 11). This study thus shed a new light on the catalytic mechanism of ribose-modifying SPOUT MTases.

Deciphering the specific contributions of individual residues to RNA binding, base flipping, or catalysis remains challenging. Regarding the RNA dynamics, a two-step model of the Tsr substrate recognition mechanism was proposed by Kuiper and Conn, in which the RNA initially binds to the enzyme before undergoing a conformational rearrangement [63]. In our mutational study, although only five RlmR variants displayed a reduction in RNA binding, most of the mutations resulted in a significant impairment of catalytic activity. Consistent with the proposed model and the crucial role of A-loop rearrangement in specific substrate recognition and activity, we hypothesize that the initial RNA docking to RlmR remains mostly unaffected by single mutations, as evidenced by the EMSA experiment, whereas the subsequent loop conformational rearrangement is disrupted, thereby impairing enzymatic function. Among these variants, the most affected were those in which residues within the SPOUT MTase motifs (N122A, R128A, and N238A), or D235, which stabilizes the U2552 base, were substituted.

The structure of the RlmR-RNA complex obtained after crystallization in the presence of SAM led to the observation of a post-reaction state in which the target U2552 is 2′-*O*-methylated in the active site, the SAM co-substrate being converted to SAH. This was rather surprising, since we would expect the products of the reaction to leave the active site following methylation. This raises the hypothesis that an additional partner or more distant regions of the RNA contribute to the release of the reaction products. As stated in the ‘Results’ section, H91 is absent in our model, likely due to a poor stabilization. This is probably caused by the crystal packing, which led us to carefully consider the positioning of H90, thereby preventing the identification of longer-range interactions between the RNA and the L30-like domains of RlmR. Conversely, in the structures of human or *T. brucei* mitoribosome assembly intermediates, other partners as well as the whole 23S rRNA are present and interact with MRM3 or mt-LAF5/6 MTases [57, 58]. This ribosomal context could explain the somewhat different positioning of the RNA, which may have been captured at a moment slightly offset from methylation. Another possibility is that the proper positioning of the target nucleotide with respect to the catalytic site residues would rely on the presence of SAM (or SAH), which is lacking in these two mitoribosomal intermediate structures. This hypothesis is supported by the absence of electron density corresponding to the RNA in the RlmR-RNA complex in the absence of SAM or a co-substrate analogue.

Most bacterial 23S modifications were shown to occur at an early stage of ribogenesis, which is in line with our results [5, 64, 65]. In contrast to RlmE, *T. thermophilus* RlmR methylates neither fully assembled 70S ribosome nor the 50S subunit but modifies RNA devoid of proteins, indicating an earlier intervention of RlmR compared to the *E. coli* enzyme, which belongs to the RFM family [11]. In agreement with this, MRM3 was captured in ribosomal assembly stages upstream of the intervention of MRM2, the human ortholog of RlmE [57, 66, 67]. The dimeric organization of MRM3 would cause severe steric clashes with the 23S rRNA if the binding occurred at a later stage of ribogenesis. This limited accessibility to the A-loop is due to its repositioning following MRM3 action. More generally, the modification of the A-loop by dimeric SpoU MTases might occur earlier than those catalyzed by RFM enzymes.

rRNA MTases are expected to play a significant role in various biological processes associated with ribogenesis and ribosome function. Indeed, even if the precise role of RNA modifications remains largely elusive, evidence suggests they are involved in the modulation of the efficiency and fidelity of translation, in antibiotic resistance, in RNA stabilization, and in oxidative stress recovery [68–70].

As it relates to U2552 modification, the absence of RlmR in *T. thermophilus* does not affect growth, ribosomal profile, sensitivity to several antibiotics, or resistance to temperature stress. At first glance, this result may seem surprising, as until recently (see the exception of *B. subtilis* below), the inactivation of any enzyme involved in A-loop modification—i.e. RlmE, MRM2, MRM3, and Spb1—was reported to have deleterious effects [14, 22, 23, 25, 27, 71]. From an evolutionary perspective, the RlmR SPOUT MTase (COG0566) described here represents a third way for forming Um2552, alongside the previously reported RFM—(COG0293) and snoRNA—based (COG1889) systems. This remarkable case of convergent evolution underscores the importance of the Um2552 modification [16, 17, 25]. Nevertheless, as with Um2552 in *T. thermophilus*, no phenotype has yet been identified in the absence of Gm2553 in the A-loop of *B. subtilis* 23S rRNA [35]. Interestingly, apart from a few cases cited above where single MTase knockout strains result in observable phenotypes, it was reported that defects in the large ribosome subunit assembly are triggered primarily by the accumulation of multiple deletions [26]. Adding to this complexity,*Clostridium* lacks any modification in the A-loop of its 23S rRNA [72]. This suggests that A-loop modification is not universally essential, revealing interspecies differences. This apparent contradiction between Um2552 significance and its dispensability makes the role of this modification even more enigmatic. Despite similarities, each species displays unique specificities in its ribogenesis pathway, whether in the partners involved, the timing of their action, or the structure of the mature ribosome, which may explain these discrepancies. Furthermore, RNA modification may play a pivotal role in enabling species to adapt to diverse environmental stresses. For instance, the large range of antibiotics encountered in its natural environment may explain the higher number of *E. coli* 23S rRNA modifications compared to an extremophilic organism such as *T. thermophilus*. Additionally, rRNA modifications in *T. thermophilus* may contribute to stabilizing the ribosomal machinery under high-temperature conditions.

In conclusion, a novel SPOUT MTase catalysing Um2552 formation in *T. thermophilus* 23S rRNA was put to light. The determination of the three-dimensional structure of the enzyme in complex with a 59-mer RNA revealed key structural features allowing this 2′-*O*-MTase to interact with its substrate and exert its catalytic activity. Further studies will be needed to deepen our understanding of protein–RNA dynamics within the ribosomal context and elucidate the exact role of RlmR in the ribogenesis process and overall physiology of *T. thermophilus*.

## Supplementary Material

gkaf432_Supplemental_File

## Data Availability

The atomic coordinates of structures described in this work were deposited in the protein databank. RlmR apoenzyme: 9MUK; RlmR-RNA-SAH: 9H1K; and RlmR-RNA-SAM: 9MUJ.
