# Supplementary material for: Structural insight into the novel Thermus thermophilus SPOUT methyltransferase RlmR catalysing Um2552 formation in the 23S rRNA A-loop: a case of convergent evolution
Source: Nucleic Acids Res. 2025 May 30;53(10):gkaf432. doi: 10.1093/nar/gkaf432 (PMC12123411; doi:10.1093/nar/gkaf432)
Supplement: gkaf432_Supplemental_File [file gkaf432_supplemental_file.pdf]

SUPPLEMENTARY DATA

Oligonucleotide name	Sequence	Purpose
TTC1712-1	GATCAAGCTTATGCGGATTGAAAGCCCGC	Amplification of *T. thermophilus ttc1712* gene
TTC1712-2	GATCAAGCTTTTATAAGGGTGCGCCCC	Amplification of *T. thermophilus ttc1712* gene
TTC1712Bam-1	CCTCTTTAGCCCCCAGGGGATCCGCAACTCCACGG	*TTC1712* mutagenesis (BamHI site)
TTC1712Bam-2	CCGTGGAGTTGCGGATCCCCTGGGGGCTAAAGAGG	*TTC1712* mutagenesis (BamHI site)
Km-1	CGTGGATCCTAAACGCGTGGAGGTGAAGCATGAATG GACCAATA	Amplification of Km thermoresistant cassette
Km-2	CGTGGATCCTCAAAATGGTATGCGTTTTGACAC	Amplification of Km thermoresistant cassette
Th23S-1	GATCTAATACGACTCACTATAGGTCAAGATGGTAAGG GCCC	Amplification of *T. thermophilus* 23S gene
Th23S-2	GATCGGTCAAGACCTCGGACGATTGGG	Amplification of *T. thermophilus* 23S gene
T2552A-1	CCCAAGGGTTGGGCAGTTCGCCCATTAAAG	*T. thermophilus* 23S gene mutagenesis
T2552A-2	CTTTAATGGGCGAACTGCCCAACCCTTGGG	*T. thermophilus* 23S gene mutagenesis
T2552C-1	CCCAAGGGTTGGGCCGTTCGCCCATTAAAG	*T. thermophilus* 23S gene mutagenesis
T2552C-2	CTTTAATGGGCGAACGGCCCAACCCTTGGG	*T. thermophilus* 23S gene mutagenesis
T2552G-1	CCCAAGGGTTGGGCGGTTCGCCCATTAAAG	*T. thermophilus* 23S gene mutagenesis
T2552G-1	CTTTAATGGGCGAACCGCCCAACCCTTGGG	*T. thermophilus* 23S gene mutagenesis
MS-1	ATGAGCCGACATCGAGGTGCC	Isolation of a specific fragment for LC-MS/MS analysis
MS-2	TTCTGAACCCAGCTCGCGTA	Isolation of a specific fragment for LC-MS/MS analysis

Table S1. Oligonucleotides used in this study.

	RlmR (apoenzyme)	RlmR-RNA-SAH	RlmR-RNA-SAM
**Pdb code**	9MUK	9H1K	9MUJ
**Wavelength (Å)**	0.98010	0.97856	0.97856
**Resolution range (Å)**	52.48 - 1.973<br>(2.044 - 1.973)	58.72 - 1.895<br>(1.928 - 1.895)	41.35 - 2.006<br>(2.078 - 2.006)
**Space group**	C 1 2 1	P 21 21 2	P 21 21 2
**Unit cell**	221.8 62.2 98.0<br>90.0 114.2 90.0	113.0 117.4 58.3<br>90.0 90.0 90.0	113.0 117.4 58.3<br>90.0 90.0 90.0
**Total reflections**	525853 (49685)	848069 (38379)	717013 (71458)
**Unique reflections**	85990 (8564)	62387 (3095)	52719 (5189)
**Multiplicity**	6.1 (5.8)	13.6 (12.4)	13.6 (13.8)
**Completeness (%)**	99.98 (99.99)	100.0 (99.8)	99.97 (100.00)
**Mean I/sigma(I)**	10.93 (0.79)	16.0 (1.6)	12.39 (0.89)
**Wilson B-factor**	36.84	32.88	34.76
**R-merge**	0.08983 (1.915)	0.104 (1.776)	0.1323 (2.41)
**R-meas**	0.09829 (2.102)	0.109 (1.853)	0.1374 (2.502)
**R-pim**	0.03938 (0.855)	0.029 (0.525)	0.03705 (0.6686)
**CC1/2**	0.999 (0.269)	0.999 (0.391)	0.999 (0.388)
**CC***	1 (0.651)	1 (0.941)	1 (0.748)
**Reflections used in refinement**	86076 (8564)	62375 (6160)	52715 (5189)
**Reflections used for R-free**	4414 (455)	3053 (295)	2588 (258)
**R-work**	0.2051 (0.3082)	0.2036 (0.3156)	0.2343 (0.3205)
**R-free**	0.2262 (0.3197)	0.2326 (0.3391)	0.2548 (0.3150)
**CC(work)**	0.951 (0.530)	0.945 (0.626)	0.941 (0.593)
**CC(free)**	0.947 (0.526)	0.934 (0.598)	0.923 (0.507)
**RMS(bonds)**	0.011	0.011	0.011
**RMS(angles)**	1.40	1.53	1.57
**Ramachandran favored (%)**	98.36	100	100
**Ramachandran outliers (%)**	0	0	0

Table S2. Data collection and refinement statistics (Statistics for the highest-resolution shell are shown in parentheses).

	MIC100 (µg/ml)	
**Antibiotics**	***T. thermophilus* WT**	***T. thermophilus ΔrlmR***
Chloramphenicol	10	10
Linezolid	20	20
Puromycin	5	5
Lincomycin	20	20

Table S3. Minimal inhibitory concentration (MIC100) of several antibiotics, allowing complete inhibition of the growth of *T. thermophilus* WT and *ΔrlmR* strains at 60°C.

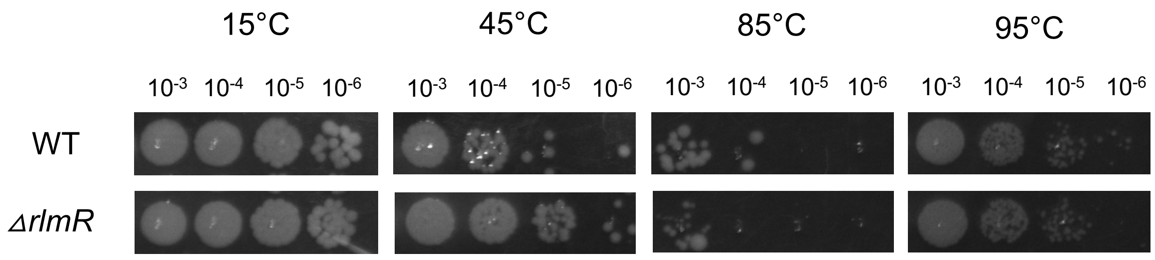

Figure S1. Growth properties of the WT (Top) and *ΔrlmR* (Bottom) *T. thermophilus* strains upon several temperature shocks. Exponential phase cells were submitted to shock temperatures at 15, 45 and 85°C during 24 h or 95°C during 10 min. The cultures were then serially diluted (10^{-3} to 10^{-6} fold dilutions), spotted onto TSB plates, and cultivated at 70°C for 24 h.

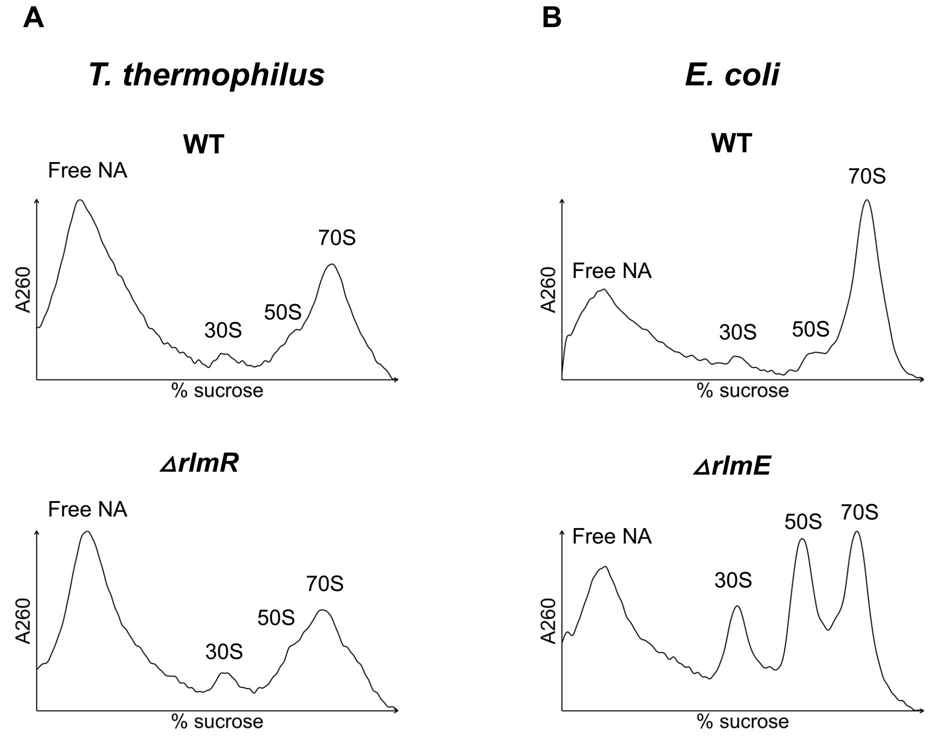

Figure S2. Sucrose density gradient profile of ribosomal subunits performed at high magnesium concentration from (A) *T. thermophilus* WT and *ΔrlmR* grown at 70°C (B) *E. coli* WT and *ΔrlmE* grown at 30°C.

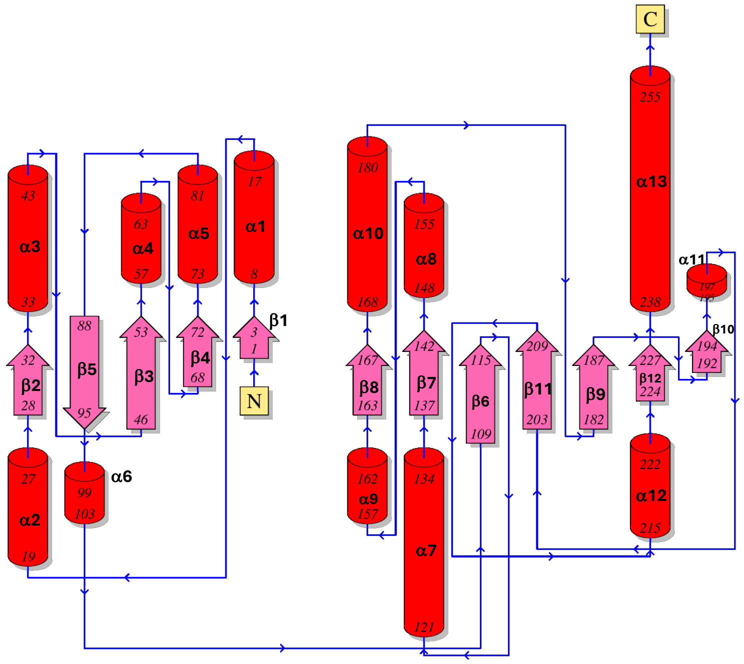

Figure S3. Topology diagram of RlmR (PDBSum).

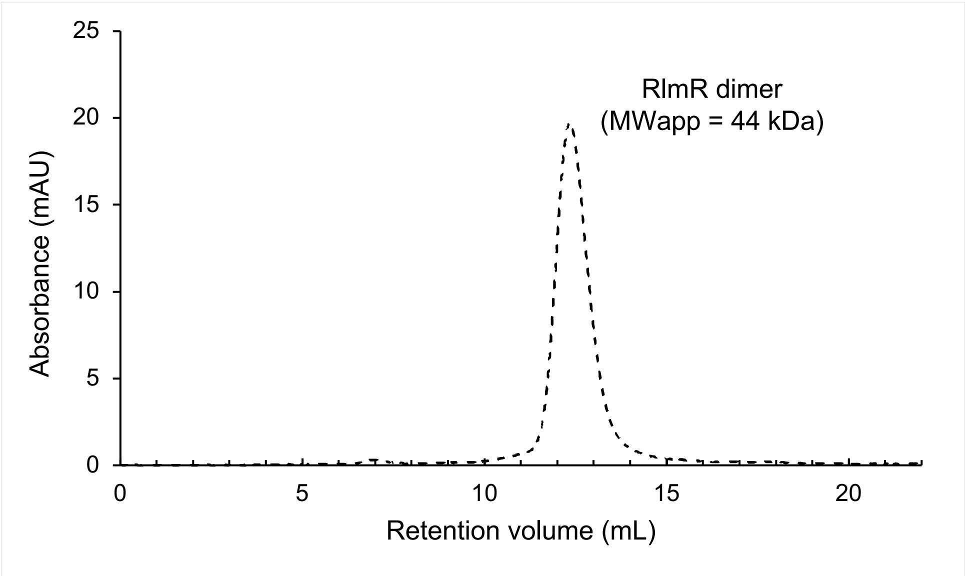

Figure S4. Size exclusion chromatography analysis of RlmR. The pure protein was loaded on a Superose 12 10/300 GL (GE Healthcare) and eluted with buffer B (50 mM Tris, 500 mM NaCl, pH 8.0). Flow rate: 0.5 mL/min. UV absorbance was monitored at 280 nm. The column was calibrated using a gel filtration standard mix resuspended in the same buffer (lyophilized mix of thyroglobulin, bovine γ-globulin, chicken ovalbumin, equine myoglobin, and vitamin B12 (Biorad)).

1 VRIESPQNPRVKALAALKERKERERTGRFLVEGRREVERALEAGLSLETLLLGPKARPED

61 RALAGGAEVLELSERALARVSARENPAQVLGVFRLPRRSLAGVTLGAAPLVLVLLGLEKP

121 GNLGAILRAADGAGADLVLVAEGVDLFSPQVIRNSTGAVFALPVYPVAEEEAARFLEEQN

181 LPLVAATPEGERLYWEGDYRGGVAFLLGAEDKGLPEAWKRRAQVRVRIPMRGRADSLNVA

241 VTAALLLYEALRQRSGGAPL

Figure S5. Amino acid sequence of RlmR. The three sequence motifs that characterize the SPOUT RNA MTase superfamily are highlighted in yellow, green, and magenta (1). The catalytic R128 is in bold and the residues which were mutated in this study are underlined.

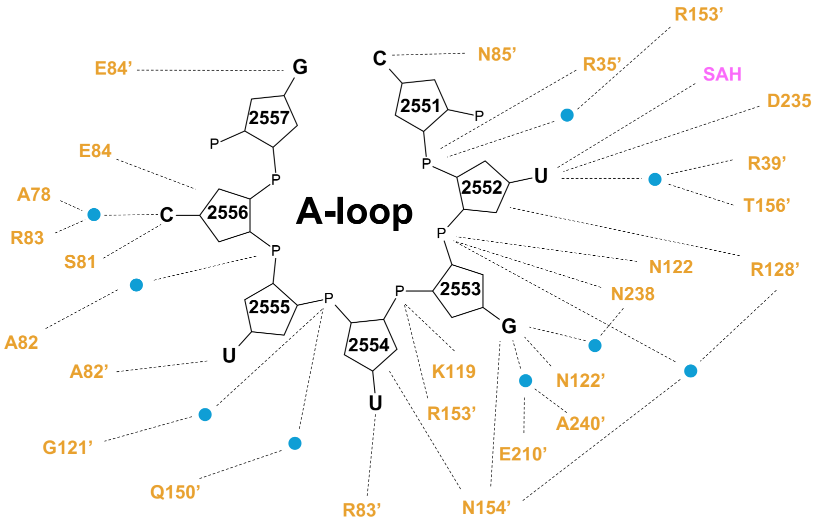

Figure S6. Schematic view of the main interactions stabilizing the A-loop in the RlmR-RNA complex. Blue spheres represent water molecules. The single quote symbol refers to monomer B.

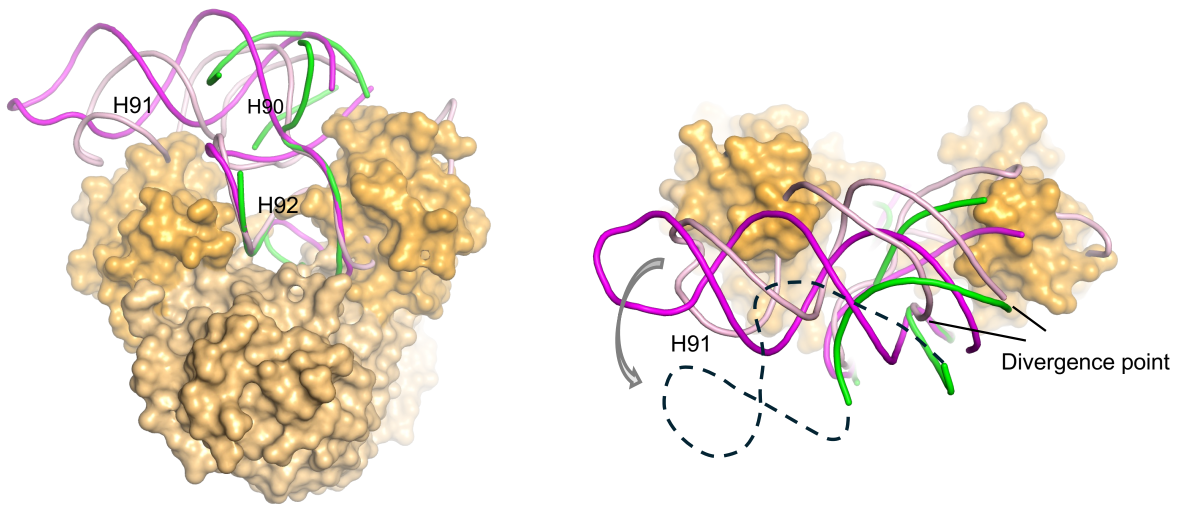

Figure S7. Side and top views of an overlay of U2552 59-mer RNA in the context of RlmR-RNA-SAH complex (green) and the corresponding residues taken from *T. thermophilus* 70S ribosome structure (pink, pdb code 4y4p) or from a model of RlmR-RNA complex obtained from AlphaFold 3 server (2) (magenta). The H92 from these structures were superimposed, showing the deviation of the H91 RNA backbone, potentially due to the crystal packing. The putative position of missing RNA residues in the RlmR-RNA-SAH complex is represented as a dashed line. The backbone of the RNAs begin to diverge where indicated.

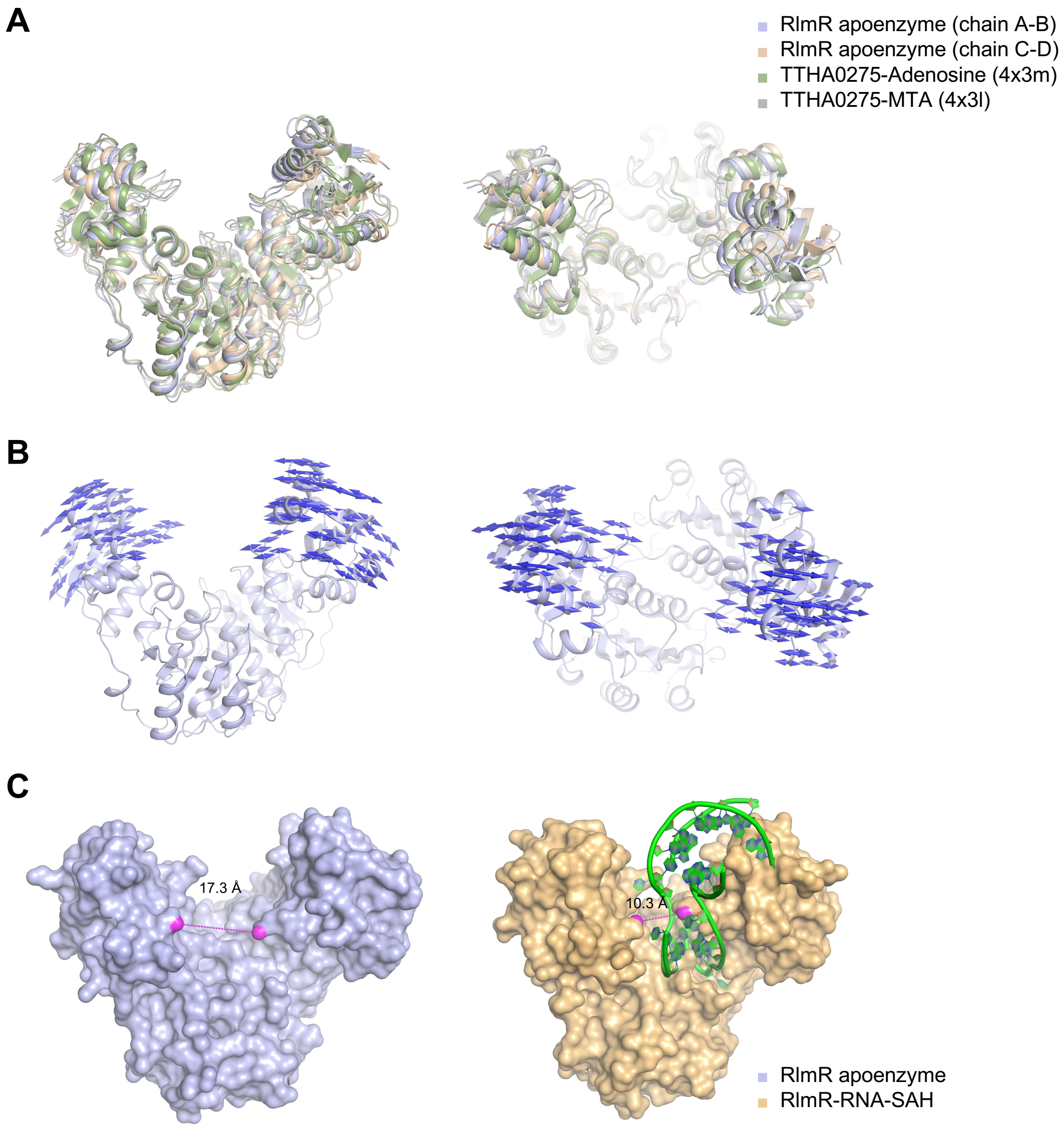

Figure S8. RlmR dynamics. (**A**) Overlay of RlmR apoenzyme and TTHA0275 (*T. thermophilus* HB8 ortholog) in complex with adenosine (pdb code 4x3m) or methylthioadenosine (pdb code 4x3l) (**B**) Elastic Network Models (ENM) analysis of RlmR apoenzyme. The normal mode analysis allows to describe the flexible states accessible to a protein. Calculations were carried out with the server elNémo (3, 4). The arrows represent the movement of the atoms associated with the lowest frequency mode of vibration. (**C**) Narrowing of the groove between both RlmR monomers following RNA binding in comparison with the apoenzyme. To illustrate this motion, the smallest distance between E19 and R84′ was measured and coloured in magenta. The RNA is coloured in green.

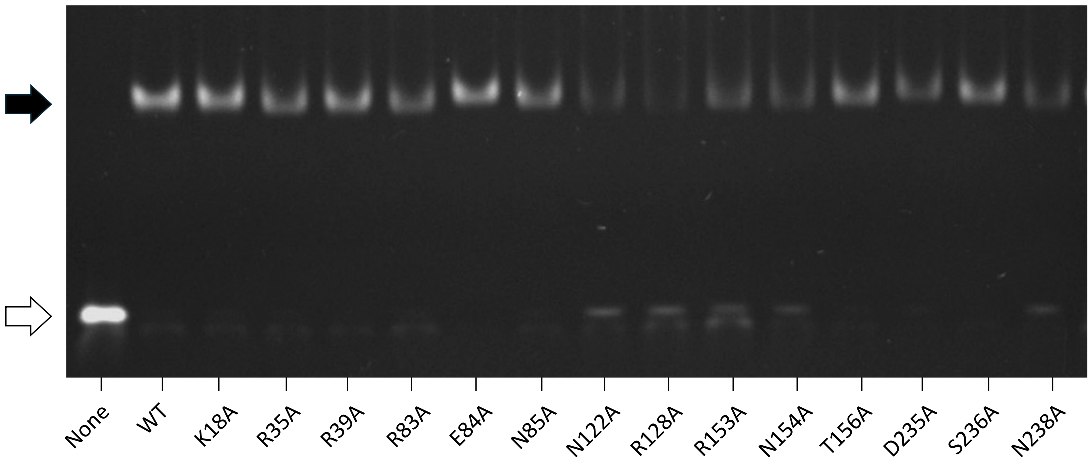

Figure S9. Electrophoretic mobility shift assay with U2552 59-mer RNA in the presence of SAH and several RlmR variants. Mutated residues were chosen based on the crystal structure of the RlmR-RNA complex. “None” refers to a blank performed in the absence of enzyme. The position of free RNA and RlmR-RNA complex is indicated with a white or black arrow, respectively.

References

1. Gustafsson,C., Reid,R., Greene,P.J. and Santi,D.V. (1996) Identification of new RNA modifying enzymes by iterative genome search using known modifying enzymes as probes. Nucleic Acids Res, 24, 3756–3762.

2. Abramson,J., Adler,J., Dunger,J., Evans,R., Green,T., Pritzel,A., Ronneberger,O., Willmore,L., Ballard,A.J., Bambrick,J., et al. (2024) Accurate structure prediction of biomolecular interactions with AlphaFold 3. Nature, 630, 493–500.

3. Suhre,K. and Sanejouand,Y.-H. (2004) ElNemo: a normal mode web server for protein movement analysis and the generation of templates for molecular replacement. Nucleic Acids Res, 32, W610-614.

4. Bauer,J.A., Pavlović,J. and Bauerová-Hlinková,V. (2019) Normal Mode Analysis as a Routine Part of a Structural Investigation. Molecules, 24, 3293.
